# The Effect of Anti-seizure Medications on the Propagation of Epileptic Activity: A Review

**DOI:** 10.3389/fneur.2021.674182

**Published:** 2021-05-27

**Authors:** Mohamed Khateb, Noam Bosak, Moshe Herskovitz

**Affiliations:** ^1^Department of Neurology, Rambam Health Care Campus, Haifa, Israel; ^2^The Rappaport Faculty of Medicine, Technion - Israel Institute of Technology, Haifa, Israel

**Keywords:** epileptic seizure, seizure propagation, seizure propagation mechanism, anti epileptic drug, classification of medications

## Abstract

The propagation of epileptiform events is a highly interesting phenomenon from the pathophysiological point of view, as it involves several mechanisms of recruitment of neural networks. Extensive *in vivo* and *in vitro* research has been performed, suggesting that multiple networks as well as cellular candidate mechanisms govern this process, including the co-existence of wave propagation, coupled oscillator dynamics, and more. The clinical importance of seizure propagation stems mainly from the fact that the epileptic manifestations cannot be attributed solely to the activity in the seizure focus itself, but rather to the propagation of epileptic activity to other brain structures. Propagation, especially when causing secondary generalizations, poses a risk to patients due to recurrent falls, traumatic injuries, and poor neurological outcome. Anti-seizure medications (ASMs) affect propagation in diverse ways and with different potencies. Importantly, for drug-resistant patients, targeting seizure propagation may improve the quality of life even without a major reduction in simple focal events. Motivated by the extensive impact of this phenomenon, we sought to review the literature regarding the propagation of epileptic activity and specifically the effect of commonly used ASMs on it. Based on this body of knowledge, we propose a novel classification of ASMs into three main categories: major, minor, and intermediate efficacy in reducing the propagation of epileptiform activity.

## Introduction

Epilepsy is a common neurological disorder, affecting over 65 million people worldwide (1). It is frequently related to cognitive and memory deficits causing significant morbidity (2, 3). In focal epilepsy, the “epileptogenic zone” was first defined as the cortical region sufficient for initiating seizures, that its removal is necessary for complete abolition of seizures (4). Later, the definition was further reduced to the minimal resection or inactivation of cortical tissue for seizure freedom (5). On the other hand, from other perspectives in the field it was proposed that the “epileptogenic zone” is not simply the “what-to-remove-area” (6). In other words, it was supposed that the epileptogenic zone may not fully overlap with the cortical area needed to be resected according to anatomo-electro-clinical correlations (6). Notably, epileptic clinical manifestations do not result solely from the activity in the seizure onset zone, but rather depend mainly on the propagation of epileptic activity to other structures, a process which in some of the cases extends to secondary generalization. Therefore, propagation of epileptic activity has a critical role in determining the severity of seizures and the resulting disability.

To date, it is still not fully understood why some focal seizures evolve to secondary generalization while others do not, even within the same patient. Pre-clinical studies suggest that the initiation and propagation of epileptic activity are dictated by different cellular and network mechanisms (7–11). The propagation process is variably affected by different anti-seizure medications (ASMs), which most commonly inhibit it, but in certain cases might even paradoxically enhance the phenomenon and hence induce secondary generalization (12). These well-known facts emphasize the importance of considering the effect of specific ASMs on this phenomenon in medical treatment planning.

The semiology regarding seizure propagation is largely region-dependent. Networks in the hippocampus, dentate gyrus, and entorhinal cortex were found to be highly correlated with seizure propagation (13–16). Other crucial regions for seizure propagation include the prefrontal cortex (and especially the orbitofrontal cortex) and others. The frontal lobe is believed to be largely involved in the propagation and especially in the inter-hemispheric spread of seizures initiated in the mesial temporal lobe (17). Importantly, specific neural circuits were identified for their crucial role in seizure propagation such as Papez's circuit. Therefore, parts of these regions are possibly candidate goals for neurostimulation to minimize the propagation of epileptic activity (18, 19).

Knowledge of propagation pathways is vital in the management of pre-operative patients. First, lateralization of epileptic activity is largely influenced by propagation. False lateralization of seizure onset in some temporal lobe epilepsy patients might be the result of abnormal or unexpected propagation patterns. In such patients, the ictal activity was demonstrated to strongly propagate to the contralateral temporal lobe but failed to propagate in the ipsilateral side misleading the determination of lateralization of seizure onset by scalp EEG (20). Moreover, it was demonstrated that variable types of propagation of ictal activities correlate with surgical outcome (21). For example, contralateral propagated ictal activity, bitemporal asynchrony, and switch of lateralization were proposed to be correlated with poor long-term seizure outcome. Hence, cautious analysis of ictal propagation patterns may provide useful biomarkers to predict surgical outcome (22, 23).

Although intervention by surgery and neurostimulation are of much interest in epilepsy management, the most common and established seizure treatment method is the use of ASMs. Therefore, in this literature review we summarize the main proposed mechanisms and the variable effects of common ASMs on seizure propagation and secondary generalization.

### Mechanisms of Propagation

In general, seizures evolve through three main phases; initiation, propagation, and termination, each of which is dictated by different mechanisms (8, 24). Seizures propagate across different brain regions *via* diverse spatiotemporal patterns. It is still unclear how exactly an epileptiform event recruits cortical circuits. Most probably, propagation is influenced by multiple mechanisms rather than a single mechanism. This statement is supported by the fact that the speed of propagation *in vivo* as well as *in vitro* can largely vary over several orders of magnitude (0.1–100 mm/s). Such a range cannot be explained by a single mechanism. According to the nucleus-shell model, focal seizures are generated from a pacemaker/seizure nucleus. Then, they propagate to other parts of the brain creating the first shell, presented clinically as a focal onset seizure with/without loss of awareness. A second shell may also develop, presented as a focal onset seizure to a bilateral tonic-clonic seizure (25).

Many models were proposed for the spatiotemporal dynamics of seizure propagation (26, 27). All seizures, even the primary generalized, are thought to propagate from initial ictal zones after originating in local microcircuits. Interestingly, this propagation is dependent on the network dynamics reflected in critical junctions or chokepoints (28). Based on data from patients with drug-resistant epilepsy, two main mechanisms for seizure propagation were suggested: wave propagation and coupled oscillator dynamics. These mechanisms coexist and can interact with each other. For example, the slow propagation of an ictal wave front was related to fast oscillations dampening the propagation. On the other hand, the fast propagation of epileptiform activities was dependent on coupled oscillator dynamics (29).

At the network level, seizure propagation is thought to be based on abnormalities of synchronization. First, the strong synchronizing activity of the epileptogenic zone largely affected the surrounding tissue (30). Second, the surrounding tissue had a limited ability to contain the incoming abnormal epileptic activity thus allowing the seizure to propagate (31). Third, antagonist activity of synchronization and desynchronization nodes caused enhancement of the propagation (32). Interestingly, the spread of epileptiform activities in neocortical slices was shown to be opposed by powerful feedforward inhibition. The strength of this feedforward inhibition was reversely correlated with the speed of spread (33). This feedforward inhibition decreased in effectiveness after repeated epileptiform events (9, 34).

GABAergic activity is largely involved in seizure propagation (35, 36). Selective parvalbumin (Pv)-positive inhibitory interneurons activation *in vitro* (by optogenetics), distant from the seizure focus, caused propagation blocking and shortening of seizure duration. On the other hand, excitation of these Pv interneurons at the epileptic focus did not prevent the ictal generation even resulting in its promotion. This is possibly achieved by inducing post-inhibitory rebound spiking in pyramidal neurons due to the increase in the high level of chloride causing local hyper-synchronization (37–39). Therefore, selective manipulations of GABAergic neurons may result in contradictory results; not only anti-epileptic but also ictogenic effects. This is believed to be mainly because of the disturbed chloride homeostasis in different phases of seizure activity (39). Furthermore, experimental observations in animal models suggested that GABAergic projections from the substania nigra to regions like the pedunculopontine nucleus and the piriform cortex are important in the process of the propagation of epileptic activity (40, 41). High frequency oscillations of somatosensory evoked potentials (SEP-HFOs) were suggested to potentially help assess the neurophysiological properties of ASMs (42). More specifically, GABAergic tone was proposed to be correlated with late component high frequency oscillations (lHFOs) (43). The number of ASMs used, in patients with focal epilepsy, correlated with the magnitude of reduction in lHFOs in the affected hemisphere possibly supporting the role of cortical GABAergic activity in the propagation of epileptic activity (42).

Interestingly, *in vitro* studies suggested that seizure propagation might potentially transform the target region into an independent epileptogenic focus that was capable of generating spontaneous and evoked seizures. This was shown to depend on NMDA receptors and excitatory shifting of the GABAergic synapses because of changes in the reversal potential of chloride (44, 45).

At the cellular level, besides previously discussed mechanisms related to GABAergic activity, additional important mechanisms were described. These include NMDA receptors upregulation and high voltage-activated calcium currents (46). Moreover, AMPA and Kainate (KA) receptors seem to be largely involved in seizure propagation as well (47–49). Increased expression of KA receptors in aberrant sprouts was proposed (47). This implies that upregulation of KA receptors promote propagation of epileptic activity. Additional possible mechanisms that were linked with seizure propagation include high extracellular potassium levels inducing regenerative potentials and enhancing synchronous activity of principle cells (37, 50–52). Other mechanisms were found to be related to the endocannabinoid system. Changes in the cannabinoid receptor availability in regions like the insula and dentate gyrus proved to be important for seizure propagation (53, 54).

### Anti-seizure Medications

In general, ASMs are believed to suppress seizure generation as well as propagation. However, the relative influence of each ASM on these processes is not fully understood. The following is a review of the possible effects of commonly used ASMs on seizure propagation Table 1.

**Table 1:** Reports on the relative efficacy of each of the discussed ASMs on the propagation of epileptic activity.

**Major effect**	**Intermediate effect**	**Minor effect**
Valporic acid (55–60)	Lamotrigine (61–64)	Carbamazepine (61, 62, 65–69)
Levetiracetam (70–75)	Topiramate (7, 76, 77)	Phenytoin (55, 67, 78, 79)
Perampanel (7, 80–82)	Barbiturates (40, 41, 83, 84)	Lacosamide (7, 61, 85)
Zonisamide (86–90)	Benzodiazepines (40, 41, 83)	
Cannabidiol (54, 91–94)		

#### Valporic Acid (VPA)

VPA is widely used for all seizure types despite the uncertainty of its mechanism of action (50). Possible mechanisms include increased GABAergic activity, suppressing excitatory neurotransmission, and modification of monoamines. VPA is considered the first-line therapy in patients with idiopathic generalized epilepsy, except in women of childbearing potential. Acting on multiple targets is possibly crucial for the generation as well as the propagation of seizures; VPA has anti-epileptic effects on several steps of seizure organization. In the model designed by Appelgate et al., VPA-treated mice did not exhibit significant propagation of seizures. All VPA-treated mice demonstrated forebrain seizures without signs of propagation to other regions (55). *In vitro* studies showed that VPA decreased the propagation speed of epileptiform events that initiated from CA3a–b and propagated bi-directionally to CA1 and CA3c (56). Similarly, in a hemoconvulsant flurothyl (FE) mice model, VPA showed an increasing effect on the threshold of FE-induced clonic convulsion and a blocking effect on the propagation (57).

Furthermore, epileptiform events in VPA-resistant patients had more extensive cortical involvement compared with events in VPA-sensitive patients (58). This possibly implies the importance of VPA in suppressing propagation. However, it is difficult to determine whether this difference in propagation resulted from the ineffectiveness of VPA in these patients, a more severe epileptic disorder, or both of them. This wider cortical involvement in VPA-resistant patients included mainly bilateral insula and frontal regions.

Using the photoparoxysmal response phenomenon, VPA had a prominent effect on duration, amplitude, morphology, and propagation pattern of visually induced epileptic events. However, VPA had a much smaller effect on the frequency of epileptic events occurrence. The conclusion was that VPA mainly reduces the spread of epileptic activity from the trigger site rather than affecting the trigger mechanism (59, 60).

Interestingly, a retrospective study of 250 patients with refractory focal seizures with impaired awareness, demonstrated that withdrawal of VPA affected seizure propagation rather than seizure onset or initiation (25).

Taken together, VPA is believed to have a major effect on the propagation of seizures.

#### Carbamazepine (CBZ)

CBZ is a first-generation ASM acting mainly on voltage-gated sodium channels, especially at the open as well as the fast inactivation phases (95, 96). It is widely used for focal as well as generalized tonic-clonic seizures. Wu et al. suggested that CBZ is more likely to affect neural excitability rather than the propagation of seizures (61). According to previous studies, sodium channels blockers and especially CBZ were proposed to raise the seizure threshold at the focus, and only exert mild effects on seizure propagation (65, 66). Similar results were reported by Arzy et al. Topographic analysis of the EEG of patients with first generalized seizure without an identified lesion in the MRI, demonstrated that CBZ administration caused a decrease in gamma-power signal (62). These findings may imply CBZ-causing modifications within local cortical circuits. The conclusion was that CBZ has an anti-epileptic effect mainly on seizure initiation rather than propagation.

On the other hand, contradicting reports strengthened CBZ's role in diminishing seizure propagation as well. For example, CBZ demonstrated a prominent effect on secondary generalized seizures in patients with focal epilepsy (67). Furthermore, CBZ was shown to inhibit seizure propagation rather than initiation in a 25-year-old woman with non-lesional epilepsy and frequent focal seizures with impaired awareness of frontal origins (68). In addition, the proposed effect of CBZ on SCN1A implies its possible negative effect on seizure propagation (69).

Interestingly, withdrawal from CBZ therapy was shown to affect seizure propagation rather than initiation (97).

Eslicarbazepine, a member of the dibenzazepine carboxamide family like CBZ and oxcarbazepine (98), was shown to increase EEG global connectivity in focal epilepsy patients, such that it was not anymore significantly lower than healthy controls (99). In other words, eslicarbazepine was shown to reverse the pathological network re-organization caused by focal epilepsy. It is difficult to estimate the exact effect of this medication on seizure initiation or propagation due to lack of evidence in the literature compared to CBZ. Nevertheless, we think the described reversal of the network re-organization may be due to suppression of local hyper-connectivity near the focus, implying that eslicarbazapine has an effect on seizure focus.

To conclude, the relative efficacy of CBZ and perhaps other members of its pharmacological group on propagation is still not fully elucidated, as there are contradicting pieces of evidence from pre-clinical as well as clinical studies. CBZ has a strong effect on seizure initiation, but it may have a smaller effect on seizure propagation.

#### Phenytoin (PTH)

Phenytoin is a first-generation ASM acting mainly on voltage-gated sodium channels. It was shown to increase the threshold for paroxysmal activity in response to electrical stimulation in a dose-dependent manner. However, it did not influence the duration or the severity of secondary generalized seizures (78). Similar results were reported in the epileptic model of flurothyl-initiated seizures in mice in which PTH lacked negative effects on seizure propagation (55). Thus, phenytoin's anti-seizure effect is thought to be more specific for initiation rather than propagation (67). Nevertheless, in some pre-clinical studies, PTH was shown to also affect seizure propagation in addition to initiation (79).

These findings suggest only a minor role of PTH on seizure propagation.

#### Lamotrigine (LTG)

Lamotrigine is a second-generation ASM. It has a relatively broad spectrum of activities and is mainly used for treating focal epilepsies with or without secondary generalizations (100). The main mechanisms of action are *via* inhibiting the activity of voltage-gated sodium and calcium channels (50, 101). Pre-clinical studies demonstrated the effect of LTG in suppressing seizure propagation in the rat amygdala kindling model (61). *In vitro* results reported by Huang et al. suggested that LTG prominently reduced the propagation of field potentials in rat PFC (63). As elaborated before, seizure propagation largely depends upon high-voltage-activated calcium currents. Consequently, by diminishing those currents, LTG is thought to inhibit propagation (46). Moreover, LTG was found to largely dampen AMPA-receptor activity in the dentate gyrus thus achieving negative effects on seizure propagation (102). This region has a crucial role in regulating the propagation of epileptiform activities in hippocampal circuits, acting as a frequency-dependent filter for incoming paroxysmal activity from the entorhinal cortex (64). Topographic analysis of the EEG of patients with first generalized seizures (non-lesional according to MRI) demonstrated that LTG administration caused a decrease in gamma power which implies there were modifications within local cortical circuits. The conclusion was that LTG, like CBZ, mainly affects seizure initiation rather than propagation (62).

Interestingly, withdrawal from LTG therapy was shown to affect seizure propagation rather than initiation (97).

Consequently, we conclude that LTG has an important effect on seizure propagation, possibly because of anti-epileptic mechanisms other than affecting voltage-gated sodium channels. However, the relative influence of LTG on propagation compared to initiation needs to be clarified by further research.

#### Levetiracetam (LEV)

The mechanism of action of LEV is largely unknown, but it was hypothesized to target neurotransmission by acting on SV2A, a pre-synaptic vesicle protein (70, 103). LEV is used for most seizure types, with a relatively low interaction profile with other medications or adverse effects. Pre-clinical studies of temporal lobe epilepsy models, including rat amygdala kindling and rat pilocarpine models, demonstrated that LEV has an effect on seizure propagation rather than initiation (61, 104). This effect is believed to stem from suppressing excitatory synaptic transmission (61, 105). Similar results were obtained by inducing non-convulsive seizures triggered by ischemic lesions in animal models. Applying LEV caused a decrease in propagation of non-convulsive seizures (71). Additional basic studies proposed that LEV induced inhibition of excessive synchronized activity between neurons without affecting normal neuronal excitability, indicating the relatively selective influence of LEV on seizure propagation (70, 72). LEV was also shown to modulate the pre-synaptic P/Q-type voltage-dependent calcium channel reducing glutamate release in critical regions for propagation like the dentate gyrus (73) and to suppress hypersynchronous activity between neurons in the CA3 areas of hippocampal slices (74).

Clinical studies showed similar results emphasizing the large influence of LEV on seizure propagation. Larsson et al. demonstrated that LEV significantly reduced the ratio of patients with epileptic spikes propagations from 22/24 to 7/15 (75). This may be due to the widespread expression of SV2A in the brain especially at regions vital for activity spreading and propagation including the dentate gyrus, entorhinal cortex, frontal cortex, several thalamic nuclei, and mesencephalon (106–108).

To conclude, clinical as well pre-clinical research largely supports the claim that LEV's anti-epileptic effect is much more prominent on seizure propagation rather than initiation possibly because of excitatory neurotransmission inhibition and modulation of regions critical for propagation.

#### Lacosamide (LCM)

Lacosamide consists of functionalized amino acids designed as an anticonvulsive medication. LCM is effective in patients with drug-resistant focal epilepsy (109, 110). LCM possesses anti-seizure as well as antinociceptive potencies (111). LCM was found to enhance voltage-gated sodium channels' slow inactivation (111–113). Slow inactivation of sodium channels is crucial in regulating firing properties and determining the basic excitable features of neurons, including the threshold of action potentials, action potential bursts, and active backpropagation of action potentials into dendritic regions (114, 115). LCM's block of the persistent sodium current (INaP) contributes to the suppression of pathological activity, without affecting normal neural activity (116). Moreover, other mechanisms were described including inhibition of carbonic anhydrase and effect on collapsin-response mediator protein 2 (CRMP-2) which is involved in axonal outgrowth and neuronal differentiation (116, 117). LCM was demonstrated to decrease inter-ictal spikes and associated HFOs in the pilocarpine models of mesial temporal lobe epilepsy (MTLE) (118). Despite its effectiveness in preventing seizures in the 6-Hz psychomotor seizure model, audiogenic seizure models, and amygdala and hippocampal kindling models, LCM's effect was low against clonic seizures induced by pentylenetetrazole, bicuculline, or picrotoxin in rodents (85, 111, 119).

Importantly, Wu et al. suggested that LCM is more likely to affect neural sensitivity rather than the propagation of seizures in a rat amygdala kindling model (61).

In a metanalysis published by Hemery et al., LCM demonstrated a significant reduction in secondary generalizations compared to the placebo. Notably, there was a trend of lower efficacy for preventing secondary generalized seizures compared with other seizures types, yet this trend was not statistically significant (7, 85). This indicates a possible inferiority of LCM in preventing propagation and secondary generalizations compared to preventing other types of seizures.

To conclude, LCM's efficiency in preventing seizure propagation is relatively low, compared to initiation.

#### Topiramate (TPM)

While the exact mechanism of action of topiramate is not fully understood, it has several potential antiepileptic effects. These include the inhibition of the post-synaptic AMPA and kainate receptors (120, 121). Importantly, this effect exists at clinically relevant concentrations (122). Additional mechanisms include blockade of voltage-activated sodium and calcium channels (123–125), and positive modulations on GABA-A receptors (126). The effect of TPM on carbonic anhydrase isoenzymes (127) is not believed to have a significant anti-seizure contribution (128). TPM was described to act both on the epileptic focus *via* increasing focal seizure threshold and inhibiting seizure propagation (76). Clinical data proposed that TPM strongly inhibited secondary generalizations in a dose-dependent manner (77). Similarly, in a metanalysis from 2014 TPM was demonstrated to significantly reduce secondary generalizations of focal seizures (7).

Taken together, TPM is thought to have a crucial effect on both seizure initiation as well as propagation. Further research is needed to explore the relative efficacy of TPM on each of the two features.

#### Perampanel (PER)

PER is an AMPA receptor antagonist that inhibits excitatory transmission by suppressing excitatory currents at the post-synaptic membrane (129). PER failed to diminish epileptic activity at focus but reduced the severity and duration of seizures compared with other ASMs such as LEV, CBZ, VPA, and others (129). These results emphasize the selective influence of PER on inhibiting seizure propagation. In the rat amygdala kindling model, PER was the only ASM checked to affect seizure propagation both near the focus as well as the distant propagation (61). Importantly, in a meta analysis from 2014, PER was demonstrated to significantly reduce secondary generalizations of focal seizures (7). PER was also demonstrated to cause a prominent reduction in the secondary bilateral synchrony in adolescents with epileptic disorders resistant to LEV (80). Interestingly, the clinical effect of PER as an add-on therapy in focal epilepsy patients was suggested to involve the glutamatergic component of thalamo-cortical projections, as demonstrated by its effect on measured early SEP-HFOs (42).

Consequently, PER is believed to have a more prominent effect on seizure propagation rather than initiation. This effect may at least partially result from the fact that AMPA receptors activity is crucial for seizure propagation (81, 82).

#### Zonisamide (ZN)

ZN is a broad spectrum ASM. Its antiepileptic efficacy was proven mainly as an adjunctive therapeutic agent in patients with focal seizures. *In vitro* studies suggested that ZN blocks repetitive firing of action potential *via* acting on voltage-sensitive sodium channels and reduces voltage-sensitive T-type calcium currents (130, 131). Moreover, the sulfamoyl group of ZN was first believed to suppress seizures like acetazolamide, through inhibition of carbonic anhydrase. However, this mechanism was later abandoned as studies showed that ZN must be administrated at doses 100–1,000 times of that of acetazolamide to achieve similar effects (132, 133). *In vivo* studies proposed that ZN suppresses the spread of focal seizures in cats from the cortex to subcortical regions (86). Data from kindled cats also suggested that ZN significantly dampened seizure propagation to subcortical regions (87). Similarly, ZN markedly attenuated seizures in regions of sensorimotor cortices and the thalamus during limbic status epilepticus, indicating its major influence on seizure propagation (88). Furthermore, ZN was shown to scavenger excess NO thus modulating cGMP. This ZN-induced intracellular cascade is largely related to the propagation of epileptic activity (89, 90).

To conclude, ZN seems to inhibit seizure propagation more than initiation.

#### Benzodiazepines (BZD) and Phenobarbiturates (PB)

GABAergic synaptic activity plays a fundamental role in epileptogenesis in general and especially in the propagation, generalization, and termination of seizures (35, 36). Experimental observations in animal models suggested that GABAergic projections from the substania nigra to regions like the pedunculopontine nucleus and the piriform cortex are important in the process of propagation of epileptic activity (40, 41). The substantia nigra (SN) was demonstrated as a crucial site of action of GABA agonist ASMs (134). GABAergic synaptic enhancement is considered the main mechanism of action of benzodiazepines and a crucial component in that of barbiturates. Commonly used BZD include midazolam, diazepam, and lorazepam. Each of them possesses a different pharmacokinetic profile. BZD as well as barbiturates modulate GABA-A receptor allosterically (135–137). Nevertheless, differences in mechanisms of activity were reported. For example, BZD increase the amplitude and the decay time of GABA-A-mediated IPSPs currents in the post-synaptic membrane but PB increase the mean open time of the chloride channel (138–140). Furthermore, PB (but not diazepam) significantly reduced the current of AMPA and kainate receptors (83). In the latter *in vitro* study, diazepam was shown to even aggravate propagating ictal-like events compared to PB that inhibited them.

Notably, GABA-A receptors' activity is modulated during status epilepticus and chronic epilepsy contributing to the resistance of ASMs. This modulation is possibly governed by factors like changes in the electrochemical gradient of chloride across the membrane and in its reversal potential (44, 45, 139, 141). In addition, changes are observed within the GABA-A receptor subunits causing alternations in the intrinsic activity of the receptor (139).

Interestingly, PB diminished the amplitude as well as the propagation of seizures in neonates (84).

To conclude, modulating GABAergic activity by medications like BZD and PB may affect seizure propagation. However, because of the possible modulation on GABAergic activity during chronic epilepsy, the total influence is complicated and sometimes unexpected.

#### Cannabidiol (CBD)

CBD is a bioactive but non-psychotomimetic constituent of cannabis sativa that is used to treat refractory epilepsy in adults and children (142–144). The exact antiepileptic role of the endocannabinoid system has not been fully clarified. CBD administration is believed to enhance the activity of inhibitory interneurons in regions critical for seizure propagation such as the dentate gyrus (54).

The earliest pre-clinical experiments suggested that CBD increased the afterdischarge threshold thus reducing the afterdischarge amplitude, duration, and propagation in electrically kindled, limbic seizures rats (145). In animal models of temporal lobe epilepsy (pilocarpine and penicillin models), CBD significantly reduced the percentage of severe generalized seizures, indicating its selective negative effect on the spread of epileptic activity (91). Moreover, CBD was demonstrated to suppress LFP burst amplitude and duration in a region-specific manner in the hippocampus (an important region for propagation and generalizations) (92). However, in the same experiments of Jones et al., CBD had no important influence on burst propagation speed, suggesting that the inhibition of seizure propagation by CBD is more likely to be *via* selectively affecting strategic or critical regions involved in propagation rather than suppressing the spread of neural activity in general. Similarly, CBD restored the normal state of excitability of the membranes of PV interneurons in the hippocampus in temporal lobe epilepsy models of kainic acid and lack of magnesium. Moreover, the administration of CBD caused a reduction of EPSPs of hippocampal excitatory pyramidal cells in a voltage-dependent manner (93, 94).

To conclude, CBD has a large impact on seizure propagation possibly by locally and selectively increasing the inhibitory tone at regions critical for seizure propagation.

## Discussion

The propagation of seizures is a crucial and interesting issue from both the basic pathophysiological and the clinical perspective. Some ASMs demonstrate strong and even selective action against seizure propagation, while others lack significant potency regarding this aspect. Taken together, it seems reasonable to consider this aspect in the medical management and prognosis of epilepsy patients. Hence, we propose to classify ASMs into three different groups: major, intermediate, and minor effect on propagation Table 1, see figure.

The first group includes levetiracetam, valporic acid, zonisamide, perampanel, and cannabidiol. There are abundant data in literature supporting their negative modulation of propagation as a major or main mechanism compared to initiation. The intermediate group includes lamotrigine, topiramate, barbiturates, and benzodiazipenes. In this group of drugs there is either sparse evidence in the literature supporting their effect on propagation, or evidence showing that they have a more potent effect on initiation rather than propagation. The third group includes carbamazepine, phenytoin, and lacosamide. This group consists of drugs that affect seizure propagation as a minor mechanism, if at all.

**Figure 1:**
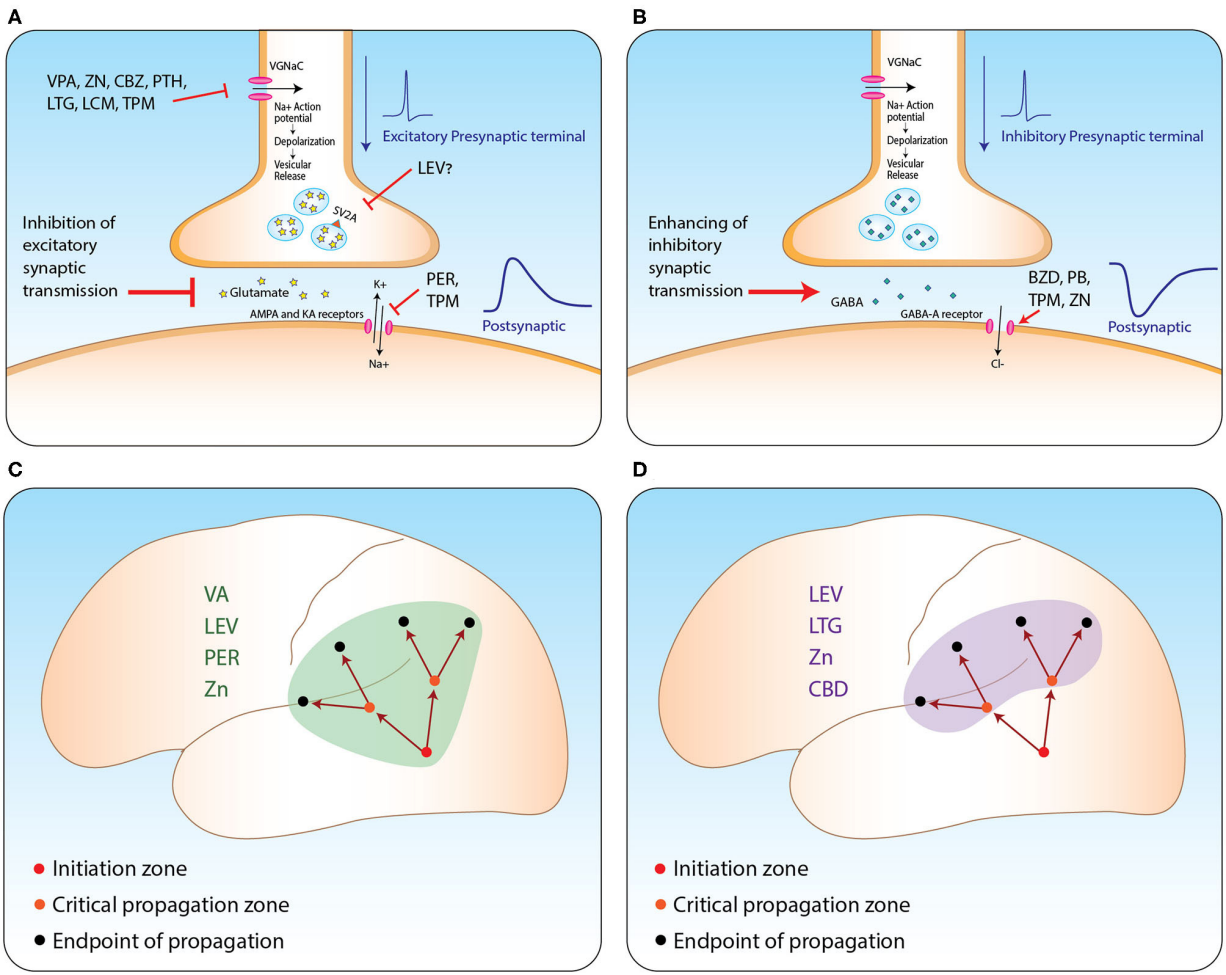
ASM mechanism from synapse to network level. **(A,B)** Suggested mechanisms of action of relevant ASMs at excitatory **(A)** and inhibitory **(B)** synapses. **(C,D)** Propagation of seizures from the initiation zone to two other types of zones: zones critical for enhancing further propagation (orange circles); and the regular endpoint of seizure zones lacking further propagation capabilities (black circles). Suppressing propagation can be achieved by two main strategies: *via* general inhibition of excitatory synaptic transmission **(C)**; or selective inhibition of the activity at critical propagation zones **(D)**. VA, valporic acid; LEV, levetiracetam; PER, perampanel; LTG, lamotrigine; ZN, zonisamide; CBZ, carbemazapine; PTH, phenytoin; LCM, lacosamide; TPM, topiramate; CBD, cannabidiol; BZD, benzodiazepines; PB, phenobarbiturates. Illustrated by Sari Eran Herskovitz.

ASMs suppress propagation of epileptic activity by two possible main mechanisms Table 2, see figure. First, they diffusely inhibit excitatory synaptic transmission in various ways. Second, some of these medications seem to have selective influence on brain regions critical for seizure propagation, such as structures within the hippocampal formation and prefrontal cortex (see Figure 1). Part of these regions are believed to further enhance propagation from the seizure initiation zone while others are described as filters or regulating gates (16, 17). For example, this filtering feature was observed in the dentate gyrus and was shown to be governed mainly by intrinsic properties of granule cells and slow inhibitory post-synaptic potentials (16). However, as the mechanisms of seizure propagation remain elusive, it is still difficult to obtain signals properly quantifying this process and thus anatomically correlating it.

**Table 2:** Proposed ASMs methods to control the propagation of epileptic activity.

**ASM**	**Inhibiting excitatory synaptic transmission**	**Selectively inhibiting regions important for propagation of epileptic activity**
Valporic acid (50)	+	-
Levetiracetam (70, 73, 103, 105–108)	+	+
Perampanel (61, 81, 82, 129)	+	-
Topiramate (120, 121, 123–125)	+	-
Zonisamide (86, 87, 89, 90, 130, 131)	+	+
Lamotrigine (50, 63, 64, 100–102)	+	+
Cannabidiol (54, 92–94)	-	+
Barbiturates and benzodiazepines	Unknown	Unknown

Future research is needed to verify our conclusions and to further investigate this important phenomenon from many aspects. For example, large retrospective or observational prospective studies following up patients for a long time may help in addressing this issue by assessing changes in propagation (extracted from clinical and anamnestic signs) resulting from raising or lowering the dosage of a specific ASM. Moreover, data from video-EEG or intra-cranial EEG monitored patients can further uncover this important issue. In these situations, abrupt or gradual cessation of ASMs is usually applied in order to exaggerate seizure's provoking as a part of routine pre-operational work-up. Finally, additional pre-clinical studies are also required to further decipher the cellular as well as the network mechanisms of ASMs in terms of effect on propagation. Possible directions include experiments using novel and valuable technologies in the field of neuroscience, such as simultaneous recordings from hundreds of neurons in different brain regions or depths using the novel neuropixels technique, two-photon calcium imaging, and more.

Taking all this into account, we conclude that ASMs may affect propagation with variable potencies enabling us to categorize them into medications with major, intermediate, and minor effects. Additionally, medications influencing excitatory synaptic transmission (such as perampanel and levetiracetam) or those selectively affecting regions strategically critical for propagating epileptic activity (like cannabidiol) are to be considered to have a more potent influence on the propagation of seizures (see Figure 1). We believe it is of great importance to be aware of this effect in order to achieve proper personalized anti-epileptic management and for improving patient quality of life.

## Author Contributions

MK initiated the idea, screened the available literature, and contributed to the creation and the design of the manuscript. MH contributed to the creation and the design of the manuscript and supervised the work. NB contributed to the overall manuscript design and final revisions. All authors contributed to the article and approved the submitted version.

## Conflict of Interest

The authors declare that the research was conducted in the absence of any commercial or financial relationships that could be construed as a potential conflict of interest.
